# Pre-Clinical Studies of Epigenetic Therapies Targeting Histone Modifiers in Lung Cancer

**DOI:** 10.3389/fonc.2013.00235

**Published:** 2013-09-09

**Authors:** Kenneth Huffman, Elisabeth D. Martinez

**Affiliations:** ^1^Hamon Center for Therapeutic Oncology Research, UT Southwestern Medical Center, Dallas, TX, USA; ^2^Department of Pharmacology, UT Southwestern Medical Center, Dallas, TX, USA

**Keywords:** HDAC inhibitors, BRD4, Jumonji demethylases, Jumonji inhibitors, EZH2 inhibitors, pre-clinical studies, lung cancer, epigenetic therapeutics

## Abstract

Treatment options for lung cancer patients have been generally limited to standard therapies or targeted interventions which involve a small number of known mutations. Although the targeted therapies are initially successful, they most often result in drug resistance, relapse, and mortality. We now know that the complexity of lung cancer comes not only from genomic changes, but also from aberrant epigenetic regulatory events. Epigenetic therapies have shown promise as single agents in the treatment of hematological malignancies but have yet to meet this expectation in solid tumors thus fostering researchers to pursue new approaches in the development and use of epigenetic interventions. Here, we review some recent pre-clinical findings involving the use of drugs targeting histone modifying enzymes both as single agents and as co-therapies against lung cancer. A greater understanding of the impact of these epigenetic compounds in lung cancer signaling is needed and further evaluation *in vivo* is warranted in several cases based on the pre-clinical activity of a subset of compounds discussed in this review, including drugs co-targeting HDACs and EGF receptor, targeting Brd4 and targeting Jumonji histone demethylases.

## Introduction

The discovery and characterization of the epigenome has produced a greater understanding of how the many different cells in an individual can have the same DNA sequence and yet develop and maintain unique phenotypes. Numerous discoveries have demonstrated specific and significant changes in the epigenetic control of cancer cells leading to the description of the “cancer epigenome” (1–3). We now recognize that altered gene expression patterns observed in cancer cells represent the cumulative output of aberrant genomic and epigenomic activity (4). Although some DNA mutations are targetable, they are not reversible (5). However, deleterious epigenetic patterns can potentially be reversed by targeting the corresponding enzymatic activities (6, 7).

Lung cancer has been primarily viewed as a disease driven by oncogenic mutations and/or oncogenic addictions (8–10). However, recent discoveries reveal a more complex view of lung pathology involving aberrant gene expression caused by cancer-specific epigenetic modifications (11). Recent non-small cell lung cancer (NSCLC) clinical trials involving epigenetic modulators have reported varying degrees of success with the most conspicuous trial published by Juergens et al. from Johns Hopkins (12). Using a low-dose regimen of entinostat and 5-azacytidine, they showed the first example of a durable response in NSCLC patients. Although the success rate in this heavily pre-treated cohort was only a modest 4%, these results should encourage new therapeutic approaches involving epigenetic agents. This mini-review focuses on new pre-clinical studies in lung cancer evaluating histone modifying drugs either as single agents or in combination with other treatment modalities.

## HDACs and Cancer

Histone deacetylases (HDACs) are enzymes responsible for removing acetyl marks from histones thereby restoring the positive charge to their lysine side chains and condensing chromatin. There have been 18 HDACs identified and they are subdivided into four major classes based on sequence homology and catalytic mechanism. HDACs have deep phylogenetic roots suggesting they evolved to regulate many proteins besides histones. Treatment of human cells with pan-HDAC inhibitors resulted in proteome-wide changes in acetylation (13). Specifically, HDACs deacetylate some of the most notorious lung cancer proteins including p53, c-myc, NF-κB, and HIF-1α (14, 15).

Although there is little evidence that somatic HDAC mutations play a role in oncogenesis, the aberrant expression and activity of HDACs is seen in many malignancies (15, 16). The global loss of acetylated H4K16 (ace-H4K16) has been observed in many tumor cell lines and tissues, and a recent study of a large number of NSCLC tissues showed that loss of acetylation for both H4K16 and H3K9 was predictive of disease recurrence (17). Furthermore, genome wide expression studies in NSCLC tissues demonstrated that both increased HDAC1 mRNA expression and reduced expression of class II HDACs were associated with poor prognosis (18, 19).

Smoking is the most causal element in the initiation and progression of lung cancers. Recent studies have reported that lung epithelia exposed to cigarette smoke condensate (CSC) exhibit global reduction of ace-H4K16 and tri-methyl H4K20 (20). Other studies show that CSC exposure causes cells to lose E-cadherin expression and undergo epithelial-to-mesenchymal transition (EMT) which can be reversed with an HDAC inhibitor (21).

### HDAC inhibitors in combination therapy

There have been numerous *in vitro*, pre-clinical and clinical studies in solid tumor models using HDAC inhibitors as single agent therapeutics with only modest success reported. Although HDAC inhibitors alter global gene expression, they may not on their own strongly activate apoptotic pathways. Moreover, unrecognized pleiotropic effects may be counteracting HDAC inhibitor induced gene expression changes. Therefore, combinatorial approaches have been favored in more recent experimental and clinical trial designs. Genome wide changes in acetylation patterns and expression output could result in changes that reverse drug resistance to more established chemotherapy regimens.

#### Vorinostat

**Figure 1:**
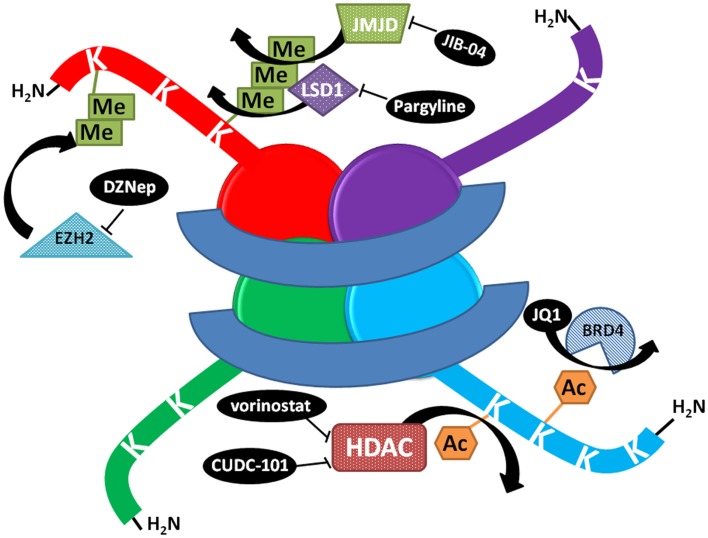
**Schematic representation of the mechanism of action of select histone modifying compounds tested on the lung cancer epigenome**. DZNep lowers EZH2 protein levels, decreasing polycomb complex activity and H3K27 methylation. JIB-04 inhibits the enzymatic activity of Jumonji histone demethylases, blocking the demethylation of tri and dimethylated histone lysines and decreasing tumor growth *in vivo*, while LSD1 inhibitors block the demethylation of monomethylated lysines. JQ1 prevents BRD4 binding to histones at acetylated lysines. HDAC inhibitors prevent the deacetylation of lysines, increasing acetylation and keeping chromatin in a more open, transcriptionally competent conformation.

Vorinostat (suberoylanilide hydroxamic acid, SAHA) is the first non-selective HDAC inhibitor (Figure 1) approved by the Food and Drug Administration (FDA), specifically for treatment of cutaneous T-cell lymphoma (22). In lung cancer models, vorinostat has shown anti-tumor activity *in vitro* but no reports of success in clinical trials exist (23, 24). NSCLC cell lines treated with vorinostat exhibit genome wide gene expression changes, including reduced expression of hTERT and increased expression of members of both the protein kinase C (PKCs) and matrix metalloproteinase (MMP) families (25, 26). The pan-genomic and pleiotropic effects of vorinostat may lead to potentially confounding issues in pre-clinical and clinical studies but may also be the catalyst needed to “re-program” the genome and increase the efficacy of co-therapies.

#### Vorinostat, EGFR TKIs, and the BIM polymorphism

EGF receptor (EGFR) tyrosine kinase inhibitors (TKIs) have shown significant success treating NSCLC patients harboring activating EGFR mutations (27), yet patients who initially show good response often develop resistance through various mechanisms. Based on significant *in vitro* and pre-clinical data, it was postulated that HDAC inhibitors may be able to reverse some of the acquired resistance mechanisms and re-sensitize cells to TKI treatment (28, 29). However, clinical trials conducted using the vorinostat/erlotinib or entinostat/erlotinib combination with NSCLC patients having EGFR activating mutations and who had already received erlotinib have shown no additive efficacy (NCT00503971) (30).

Although most patients carrying EGFR activating mutations respond to TKIs, about 20% exhibit intrinsic resistance. A report coming from Kanazawa University concerns the use of vorinostat to reverse TKI resistance in patients harboring an intron deletion polymorphism in BCL2-like 11 protein (BIM) (31). The BIM protein, specifically the BH-3 domain, which is preferentially removed by splicing in the polymorphic transcript, is necessary for TKI sensitivity (32). One NSCLC cell line exhibiting TKI resistance and the BIM polymorphism was treated with vorinostat resulting in restored expression of the non-mutant transcript and increased apoptotic response to gefitinib. These findings were replicated *in vivo*. When vorinostat was added to the gefitinib protocol, tumors with the BIM polymorphism showed up-regulation of wt BIM and regressed almost completely without notable adverse effects. The specific reason(s) why vorinostat preferentially induced the wt BIM transcript in BIM polymorph xenografts are unknown. A comparison of BIM mutations across the NSCLC demographic reveal that the BIM polymorphism is seen only in East Asian populations, but is not noted in either Caucasian or African groups (33).

#### Vorinostat with radiotherapy

Approximately half of all lung cancer patients will receive some kind of radiotherapy (RT), either by external beam or brachytherapy. RT is effective, but not often curative. Significant effort has been dedicated to increasing RT efficacy since this is often indicated for patients who cannot undergo surgical intervention. Although we are not aware of any published clinical or xenograft data utilizing only RT and HDAC inhibitors in lung cancer models, some interesting observations were made during an *in vitro* study using NSCLC cell lines (34). The authors noted a significant reduction in cell viability using RT/vorinostat co-treatment and the response appeared to be mediated by increased p53 expression. Moreover, they noted the accumulation of ace-K382 on wild type p53 and a p53-dependent reduction of c-myc expression. They confirmed this result by showing that a p53 null NSCLC line shows no additive or synergistic response with co-treatment and that c-myc expression levels do not change. It will be interesting to see whether or not the encouraging results for this RT/HDAC inhibitor co-therapy are repeatable with other wild type p53 NSCLC lines and in xenograft experiments. Of further interest would be examining the success of co-treatment with non-deletion p53 mutations since these mutations are very common in NSCLC. Currently, there is a clinical trial using RT in combination with cisplatin, pemetrexed, and vorinostat in NSCLC patients with non-resectable, locally advanced disease with results due in late 2013.

### Single compound, multi-target inhibitors

The use of some HDAC inhibitors with other modalities in a multi-targeting schema has shown success in pre-clinical and clinical studies. However, the use of a multi-drug treatment regimen can result in pharmacokinetic concerns and additive toxicities. A new approach seeks to accomplish multi-targeting within a single multi-functional compound. Here we talk about a new drug which combines an HDAC inhibitory functionality with an activity against HER2 kinases.

#### CUDC-101

This rationally designed molecule, synthesized by Curis, seeks to combine inhibition of class I and II HDACs (Figure 1) along with modulation of EGFR and HER2 kinases (35, 36). In an effort to overcome TKI resistance after first line treatment, the molecule CUDC-101 incorporates the HDAC inhibitory hydroxamic acid structure with the phenylaminoquinazoline moiety of the efficacious TKIs. The authors show that the activity of CUDC-101 in 10 NSCLC cell lines was generally more effective than a combination of vorinostat and erlotinib. Their results do include CUDC-101 treatment using two EGFR mutant cell lines H1975 (activating L858R and EGFR T790M resistance mutation) and HCC827R (EGFR activating deletion with acquired TKI resistance). Treatment of the erlotinib resistant H1975 cell line with CUDC-101 gives an IC_50_ of 500 nM and is able to significantly reduce the expression of EGFR. The HCC827 cell line normally exhibits nanomolar sensitivity to erlotinib, but in their study, they use an erlotinib resistant subculture (HCC827R, erlotinib IC_50_ = 7.5 μM) and again show nanomolar sensitivity to CUDC-101. IHC analysis of CUDC-101 treated HCC827 xenograft tumors showed a reduction in phospho (p) EGFR and pHER2 as well as a reduction in Ki67 and an increase in caspase-3. Interestingly, CUDC-101 treatment of the MET amplified NSCLC cell line H1993 showed that the compound was able to reduce p-AKT and p-MET. It is believed that an increase in p-Akt and p-Met may play a role in the establishment of TKI resistance suggesting the potential importance of this result (37).

Of note, Curis has also produced a second multi-target molecule, CUDC-907, which also employs the same HDAC inhibitory hydroxamic acid functionality utilized in CUDC-101, but places it into a core morpholinopyrimidine scaffold structure shared by PI3K inhibitors (38). This molecule is not as far along in the developmental pipeline as its CUDC-101 counterpart, but has also shown some promising activity in NSCLC cell lines.

## Up and Coming Compounds Targeting Other Histone Modifiers

In contrast to the many reports which have been published describing the activity of HDAC inhibitors as single agents or in therapeutic combinations in pre-clinical models of lung cancer, little is known regarding the activity of other classes of small molecules targeting other histone modifying enzymes. Several new agents targeting histone methyltransferases, histone demethylases, histone kinases, and chromatin remodelers have been developed, but few of these have been evaluated in lung cancer. In this section, we highlight findings on a modulator of polycomb complexes affecting EZH2 activity called DZNep, on modulation of the histone demethylase LSD1, on an inhibitor of the Jumonji histone demethylase family named JIB-04, as well as on the activity of JQ1, a Brd4 inhibitor targeting bromo domains.

### DZNep and polycomb modulation

EZH2 is a H3K27 methylase integral to the polycomb repressive complex which negatively regulates gene transcription. In lung cancers, this enzyme has been found to be upregulated both at the mRNA and protein levels and its downregulation appears to be therapeutically beneficial (39–42). The *S*-adenosylhomocysteine hydrolase inhibitor 3-Deazaneplanocin A (DZNep), was found by Tan et al to be a modulator of polycomb function through downregulation of the EZH2 protein (Figure 1) and other components of the polycomb complex (43). Two recent studies have reported on DZNep’s activity against lung cancer.

Kikuchi and colleagues (44) showed that DZNep inhibits the growth of four NSCLC lines as measured by MTS viability and soft agar colony formation assays. The compound appears to have little effect on cell cycle kinetics and is consistent with results showing decreased cell proliferation after knockdown of EZH2 (44). A separate study has explored the re-expression of MAGE genes by DZNep and finds that either downregulation of EZH2 or DZNep treatment can result in decreases in H3K27 trimethylation on MAGE regulatory regions and subsequent increased MAGE expression. This increase expression is further potentiated by treatment with DNA methyl transferase inhibitors. Higher levels of MAGE expression make the tumor cells more immunogenic and thus susceptible to T cells (45). Thus, at least in these two studies DZNep shows some pre-clinical activity against lung cancer that is mediated by the inhibition of polycomb enzymatic activity. Combination studies and further *in vivo* work will be necessary to define the potential of DZNep against lung tumors.

### LSD1

The amine oxidase LSD1 is a histone demethylase acting mainly on di- and monomethylated H3K4 but with potential to also demethylate H3K9 di- or monomethylated substrates as part of regulatory complexes (46, 47). In lung cancer patient samples, Hayami et al have found overexpression of LSD1 compared to benign matched tissues both by microarray and qRTPCR (48). RNAi mediated knockdown of LSD1 in lung cancer lines A549, LC319, and SBC5 resulted in reduced cell numbers and modest changes in cell cycle distribution (lower S phase and higher subG1 populations), suggesting LSD1 may contribute to lung cancer proliferation. In furtherance of this relationship between LSD1 and lung cancer proliferation, Lv and colleagues used IHC, immunoblot, and qRTPCR to show LSD1 was overexpressed in 80 NSCLC tumors when compared to 20 normal patient samples. Importantly, patients with high expressing tumors had poorer survival as analyzed both by RNA and protein levels (49).

Treatment of lung tumor lines A549 and H460 (which both express LSD1 with markedly higher levels in H460) with pargyline, a general blocker of amine oxidase activity (Figure 1), resulted in growth inhibition mirroring the effects seen in LSD1 knockdown experiments. Moreover, overexpression of LSD1 in A549 not only increased their proliferation but also their invasive potential as show in Matrigel invasion assays. Knockdown of LSD1 in the higher expressing H460 cells reduced their migration (49). Taken together, these data implicate LSD1 as a potential epigenetic target for lung cancer treatments.

Following on these reports, Hazeldine et al. synthesized a series of low molecular weight amidoximes with *in vitro* activity against LSD1 and tested them in the lung tumor line Calu-6. They observed reactivation of a few silenced tumor suppressor genes following treatment but did not evaluate growth inhibition (50).

### JIB-04 and other LDR hits

Using a broad cell-based assay (the LDR or locus depression assay), we identified a number of potential epigenetic modulators with activity against lung cancer cells (51, 52). In particular, a series of 8-hydroxyquinolines, which scored as hits, demonstrated potent anti-proliferative activity in H358 cells and had the ability to reactivate the expression of the silent CDH13 gene. The compounds in these series, however, did not show activity as HDAC or DNA methyltransferase inhibitors suggesting they may target other epigenetic components. These epigenetic targets may be cancer-specific since at least two hits from the 8-hydroxyquinoline series demonstrated selectivity for lung tumor lines versus patient-matched immortalized normal lung epithelial cells (52). Since this study, several groups have suggested that hydroxyquinolines may be inhibitors of histone demethylase enzymes, including the iron dependent Jumonji family (53, 54).

We have recently characterized both *in vitro* and *in vivo*, the anti-cancer activity of a new inhibitor of Jumonji histone demethylases identified in our screen. This compound, JIB-04, shows potent and selective anti-proliferative activity both in cultured cells and in mouse xenograft lung cancer models. The compound affects transcriptional programs in lung cancer cells without affecting normal cell transcriptional patterns. JIB-04 is the first epigenetic modulator targeting demethylases to show *in vivo* activity (Figure 1) and is effective administered either intraperitoneally or by gavage (55). A number of studies have noted deregulation of members of the Jumonji family of histone demethylases in lung malignancies (56–61) and investigation in this area actively continues and is of high interest.

### JQ1

BET inhibitors have recently generated much excitement. These compounds, exemplified by JQ1, block the association of BET proteins such as BRD2, 3, and 4 with acetylated histones on DNA (Figure 1). In c-myc-driven tumors, such as those in certain hematologic malignancies, treatment with JQ1 results in the downregulation of myc and its target genes (62). Although little precedent exists supporting a critical role for BET proteins in lung cancer, the ability for JQ1 to down-regulate myc led to testing this compound against myc-driven lung adenocarcinomas.

Lockwood et al therefore evaluated the response of lung adenocarcinoma cells to JQ1 inhibition in dose response studies over 72 h exposure and defined a subset of sensitive cell lines that were growth inhibited. Although the IC_50_ values were not highly potent (average 1.2 μM) the response of sensitive cells appears to be related to the downregulation of the FOSL1 transcription factor and its target genes, independent of effects on c-myc levels (63). No animal work has yet been performed to evaluate the effects of JQ1 in *in vivo* models of lung cancer, but there is the possibility that FOSL1 may be a mediator of response in lung adenocarcinomas.

## Perspective and Conclusions

We have briefly summarized selected results from recent work exploring epigenetic therapies at different stages of development for efficacy in lung cancer models. Although a number of epigenetic modulators have not yet been evaluated against lung cancer (64), we hope to encourage more in depth studies of therapies which have already shown some promise in pre-clinical studies. First, a broader exploration of NSCLC cell line sensitivity to these agents is needed and should include all possible histotypes and oncogenotypes. This type of panel study will hopefully lead to the discovery of biomarkers informing epigenetic drug choices. Second, it is our hope that labs will continue to take these *in vitro* leads through an evaluation process that will include determinations of efficacy in xenograft and genetically engineered animal models. More importantly, we hope that the “give the maximum tolerated dosage” paradigm will be re-evaluated when it comes to the epigenetic modifiers. Although we may yet discover lung cancer’s silver bullet among epigenetic drugs, we believe it will be necessary to take a more rigorous look at dosing regimens and possibly more importantly, the timing of administration for these types of agents. If the goal is to re-program the cancer genome using epigenetic modifiers, the effects may not be observed in short term, high dose evaluations but may require longer term treatments at moderate dosing.

## Conflict of Interest Statement

The authors declare that the research was conducted in the absence of any commercial or financial relationships that could be construed as a potential conflict of interest.
